# Systemic Biologic Treatment for Psoriasis in Elderly Patients

**DOI:** 10.3390/jcm14134779

**Published:** 2025-07-07

**Authors:** Sapir Glazer Levavi, Ronny Maman, Shany Sherman, Daniel Mimouni, Lev Pavlovsky

**Affiliations:** 1 Division of Dermatology, Rabin Medical Center, Beilinson Hospital, Petach Tikva 49100, Israel; 2 Faculty of Medical and Health Sciences, Tel Aviv University, Tel Aviv 69978, Israel

**Keywords:** psoriasis, elderly, biologic treatment

## Abstract

Approximately 3–13% of patients with psoriasis are first affected after age 60 years. Numerous studies have investigated the use of biologic agents for the treatment of psoriasis, but the routine exclusion of elderly adults from clinical trials has limited our knowledge about their effects in this age group. **Background/Objectives**: This study aimed to investigate the efficacy and safety of biologic drugs for the treatment of psoriasis in elderly patients. **Methods**: A single-center-based retrospective cohort study design was used. A total of 149 elderly patients with psoriasis were divided into two groups by age at initiation of biologic treatment: <65 years (adult-start group) and >65 years (elderly-start group). Data on patient characteristics were collected and analyzed, and drug survival was evaluated. **Results**: Demographics, comorbidities, and treatment turnover were similar in the adult-start and elderly-start groups. Drug survival of adalimumab as first-line treatment, and of guselkumab in any treatment sequence, was significantly better in the elderly-start group (*p* = 0.029 and *p* = 0.032, respectively). There was no between-group difference in drug safety, as reflected in hospitalizations for infection or death. **Conclusions**: Biologic treatments for psoriasis demonstrate both efficacy and safety in elderly patients. Some agents exhibited better drug survival when initiated after age 65 years.

## 1. Introduction

Psoriasis is a chronic immune-mediated inflammatory condition [1]. Its prevalence in adults ranges between 0.51% and 11.4% [2], and it has a strong age-related association [3]. Approximately 3–13% of patients are affected after age 60 years, among whom 15% have moderate to severe disease [4,5]. However, managing psoriasis in the elderly poses unique challenges owing to their higher burden of comorbidities and polypharmacy. In addition, the toxicity, systemic effects, and limited adherence associated with traditional anti-psoriatic therapies may be exacerbated in older patients due to metabolic alterations [4,6]. The chronic nature of psoriasis necessitates prolonged treatment, highlighting the importance of a tailored approach [7]. Thus, it is imperative that we deepen our understanding of the available treatment options in the elderly patient population.

Numerous studies have investigated the use of biologic agents for the treatment of psoriasis, but older adults are often under-represented in clinical trials [8]. The objective of the present study was to assess the efficacy and safety of biologic therapy in elderly patients with psoriasis based on demographic and clinical characteristics and to evaluate drug survival [9].

## 2. Materials and Methods

A retrospective study was conducted at Rabin Medical Center, the largest hospital of Clalit Health Services, Israel’s largest healthcare provider. The study was approved by the Institutional Review Board (IRB) of Clalit Health Services General Management in Rabin Medical Center—Beilinson Hospital (approval #0006-13). The procedures followed in this study adhered to the tenets of the Declaration of Helsinki. Due to its retrospective and anonymized design, the IRB waived the requirement for patient informed consent.

According to Israeli regulatory guidelines for the treatment of psoriasis, biologic agents may be initiated under one or more of the following conditions: involvement of more than 50% of body surface area (BSA), a Psoriasis Area and Severity Index (PASI) score greater than 50, or clinically significant involvement of sensitive areas such as the face, neck, body flexures, genitalia, and palms or soles. In addition, patients must have previously failed at least two standard systemic therapies, including phototherapy.

While these thresholds are more stringent than those commonly used in other countries (e.g., PASI ≥ 10), they reflect the formal policy adopted by the Israeli Ministry of Health and the major health maintenance organizations. A substantial proportion of patients in our cohort met eligibility based on special area involvement, which may result in significant functional impairment and diminished quality of life, even when the overall PASI score is below the numeric threshold. In such cases, formal scoring systems such as the Nail Psoriasis Severity Index (NAPSI), Psoriasis Scalp Severity Index (PSSI), or Physician Global Assessment (PGA) are not routinely required. Rather, qualitive measures are used, and treatment eligibility is typically based on documented evidence of involvement of these areas.

The internal guidelines of Clalit Health Services stipulate that tumor necrosis factor (TNF) inhibitors and apremilast should serve as the first-line options for biologic or small-molecule therapy. Other biologic drugs, such as interleukin (IL)-17 and IL23 inhibitors, are recommended as second-line therapy.

Data were collected between 2002 and 2022 at Beilinson Hospital. Patients aged > 65 years at the time of data collection were included if they had dermatologist-confirmed psoriasis and had received biologic therapy or apremilast. Importantly, patients were stratified based on their age at initiation of biologic treatment, irrespective of their current age, into two groups: before age 65 years (adult-start group) and at age 65 years or later (elderly-start group).

Inclusion criteria were: (1) age > 65 years at the time of data collection, (2) a dermatologist-confirmed diagnosis of psoriasis, and (3) treatment with a biologic agent or apremilast.

Exclusion criteria included previously approved therapies that have been withdrawn from the market, including Efalizumab and Alefacept.

For drug survival analysis, treatment was considered terminated if no prescription was claimed for at least 270 days for TNF inhibitors or for at least 365 days for any other biologic agent. Patients were censored in cases of death, registration at a different healthcare provider, emigration from Israel, or end of the follow-up period. Drug survival was calculated and compared between two study groups to evaluate treatment efficacy. Additional comparisons were made between the adult-start group and subgroups of the elderly-start group (ages 65–70, 70–75, and above 75 years) to further delineate treatment efficacy in the elderly population. Lack of response of first biologic treatment was defined as a PASI improvement of less than 50%, typically evaluated after at least 16 weeks of treatment. The results were analyzed using descriptive statistics. Categorical variables (sex, obesity, diabetes, hyperlipidemia, hypertension, history of malignancy, psoriatic arthritis, inflammatory bowel disease, persistent disease, and failure of previous standard systemic treatment) were described in terms of prevalence and percentages and compared between groups using chi-square test. Continuous variables (age, weight, body mass index, and disease severity/response to treatment, which was analyzed as PASI and delta PASI, respectively) were assessed for normal distribution using histograms. As means were not normally distributed, these variables were described in terms of median and interquartile range and compared between groups using the Mann-Whitney test. The number of hospitalizations and the total number of biologic treatments for each patient were compared between groups using the negative binomial distribution. Time from initiation of biologic treatment to first hospitalization, duration of first biologic treatment, and duration of all other biologic treatments were compared between groups using Kaplan-Meier survival analysis.

All statistical tests were two-sided, and a p level of less than 0.05 was considered statistically significant. Data analyses were generated with IBM-SPSS for Windows, version 29 (Armonk, NY, USA, 2022).

## 3. Results

**Figure 1:**
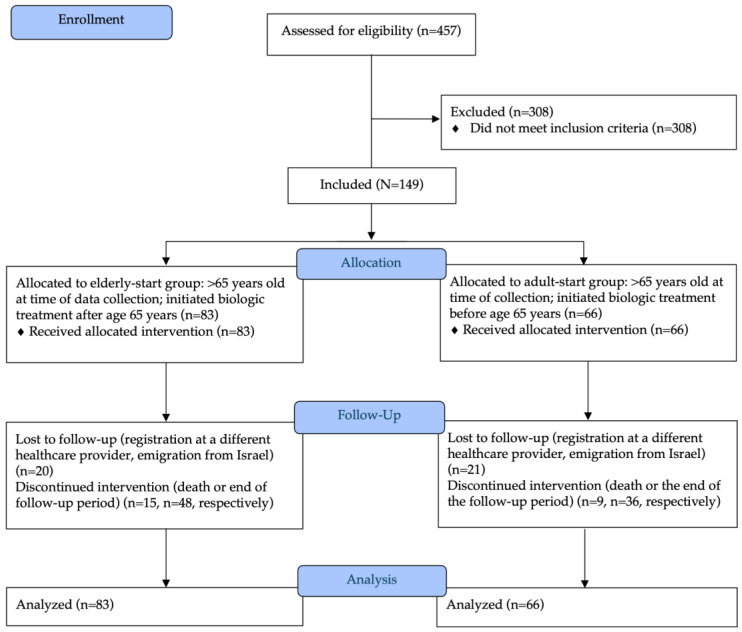
Study Enrollment process.

Study enrollment and the baseline demographic and clinical characteristics of each group are presented in Figure 1 and Table 1. A total of 457 patients with psoriasis received biologic treatment in 2002–2022, of whom 308 did not meet all the inclusion criteria. The remaining 149 patients, all aged 65 years or more at data collection, formed the study cohort. They included 83 patients who started biologic treatment at age 65 years or more (elderly-start group) and 66 who started biologic treatment before age 65 years (adult-start group). The elderly-start group was further divided into three subgroups by age at which biologic treatment was initiated: 65–70 years (*n* = 50), 70–75 years, (*n* = 17), and more than 75 years (*n* = 16).

The Figure 1 illustrates the study enrollment process, including the total number of patients assessed for eligibility, exclusion, and the final number of participants included in the analysis. The flow diagram also highlights the allocation of participants into study groups and their progression through the study, including follow-up and analysis stages. Note: The elderly-start group was further divided into three subgroups based on the age at which biologic treatment was initiated: 65–70 years (*n* = 50), 70–75 years (*n* = 17), and >75 years (*n* = 16).

### 3.1. Efficacy of Biologic Treatment

Patients’ characteristics are presented in Table 1. Overall, our study revealed no notable differences in the prevalence of comorbidities between the elderly-start group and the adult-start group.

Initial disease severity and response to treatment were assessed using PASI—results are shown in Table 2.

The initial mean PASI score was 21.3 in the elderly-start group and 22.7 in the adult-start group. After the first biologic treatment, the PASI response (delta PASI) was less than 50 in 38 patients (50.7%) in the elderly-start group and in 25 patients (43.9%) in the adult-start group, indicating a failure of first-line treatment. After the final biologic treatment, the PASI response was less than 50 in only 4 patients (4.8%) and 18 patients (29%), in the elderly-start group and the adult-start group respectively. Additionally, 47 patients (60.3%) in the elderly-start group had no detectable disease (PASI 100) compared to 26 patients (41.9%) in the adult-start group. The response to the final biologic treatment was significantly better in the elderly-start group (*p* = 0.003).

**Figure 2:**
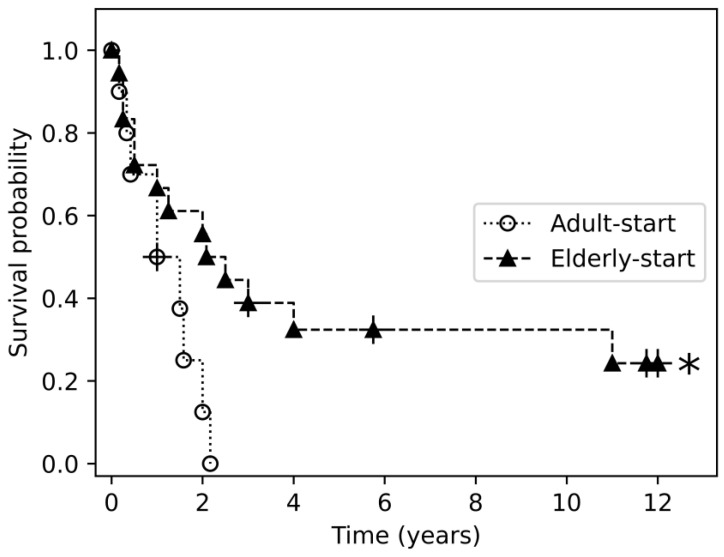
Drug survival of adalimumab as first treatment in the elderly-start and adult-start groups. The asterisk symbol (*) denotes the group in which a statistically significant survival benefit was observed.

**Figure 3:**
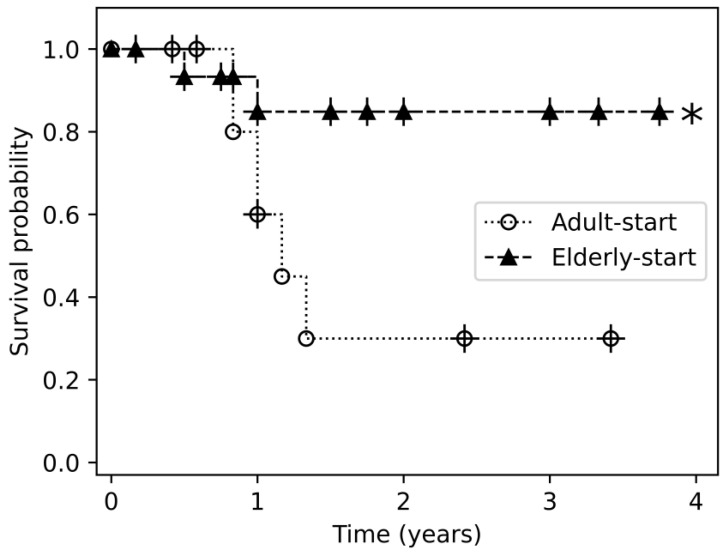
Drug survival of guselkumab in in any sequence of treatments in the elderly-start and adult-start groups. This figure shows the Kaplan-Meier survival curve representing drug survival over time. The *x*-axis displays time (e.g., years), and the *y*-axis indicates cumulative drug survival probability. Each line corresponds to a different group, with markers indicating specific data points (e.g., triangles for the elderly-start group and circles for the adult-start group). Censored events are marked with vertical tick marks, indicating patients who discontinued treatment due to death, registration with a different healthcare provider, emigration from Israel, or the end of the follow-up period. Triangles represent the elderly-start group and circles represent the adult-start group. Censored events are marked with vertical tick marks. In some cases, a triangle or circle may appear with a vertical line through it—this simply indicates that a censored event occurred at the same time point and is not of additional significance, the asterisk symbol (*) denotes the group in which a statistically significant survival benefit was observed. Key comparisons between groups are shown, highlighting significant differences in drug survival rates, with guselkumab in any treatment sequence showing better drug survival in the elderly-start group (*p* = 0.032).

Drug survival was evaluated with the Kaplan-Meier method for 9 agents: TNF inhibitors etanercept, adalimumab, and infliximab; phosphodiesterase (PDE)-4 inhibitor apremilast; IL17 inhibitors secukinumab and ixekizumab; and IL23 inhibitors ustekinumab, guselkumab, and risankizumab. Significant between-group differences in the duration of treatment were observed with certain agents (Figure 2, Figure 3 and Figure S1).

Among patients receiving adalimumab as the first biologic drug, the duration of treatment was longer in the elder-start group (*p* = 0.029). The difference from patients in the adult-start group reached trend-level significance on comparison with the 65–70-year-old subgroup (*p* = 0.07). Among patients who received guselkumab, the duration of treatment was longer in the elderly-start group (*p* = 0.032), and specifically in patients more than 75 years old (*p* = 0.046).

### 3.2. Patterns in Biologic Treatment

TNF inhibitors were the most common agents used. Etanercept was the primary choice for 52 patients (62.6%) in the elderly-start group and 47 patients (71.2%) in the adult-start group, followed by adalimumab and then ustekinumab, as shown in Table S1. Etanercept was given for the longest average duration: 2.73 years in the elderly-start group and 3.44 years in the adult-start group. The average duration of treatment was much shorter for newer agents, which were most often administered as advanced lines of therapy.

The average number of total biologic treatments received per patient was 2.5 in the elderly-start group and 3.4 in the adult-start group, with no significant between-group difference. All agents administrated in each treatment line are summarized in Table S2.

### 3.3. Safety

In the elderly-start group, 14 patients were hospitalized for one or more infections, for a hospitalization rate of 16.8%. The number of hospitalizations per patient ranged from 1 to 4, totaling 26 (31.3%). The average annual rate was 0.39, and the average time to the first hospitalization was 3.9 years. In the adult-start group, 11 patients were hospitalized for one or more serious infections, for a hospitalization rate of 16.6%. The number of hospitalizations per patient ranged from and 1 to 3, totaling 16 (24.2%). The average annual rate was 0.2, and the average time to first hospitalization was 7.6 years. Although there was a tendency for more hospitalizations in the elderly-start group compared to the adult-start group, this difference did not reach statistical significance (*p* = 0.071, IRR = 1.928).

The most common type of infection overall was pneumonia, documented in 9 patients (6% of all patients in both groups). Six patients in the elderly-start group acquired pneumonia under treatment with adalimumab, ustekinumab and infliximab, and 3 patients in the adult-start group acquired pneumonia under treatment with ustekinumab and adalimumab. Other infections leading to hospitalizations are shown in Table S2. Since no significant difference was observed in the overall infection rates between the groups and given the small number of events for each specific infection type, no separate statistical analyses were conducted for individual infections.

Fifteen patients (18.1%) in the elderly-start group died compared to 9 (13.6%) in the adult-start group, with no statistically significant between-group difference. Causes of death in the elderly-start group were infection in 8 patients, followed by malignancy and liver disease in 2 patients each and confusion in 1 patient. In 2 patients, the reason for death was unknown. In the adult-start group, 2 patients died of malignancy and 1 patient each died of liver disease, heart disease, chronic obstructive pulmonary disease, and hemorrhage. In 2 patients, the cause of death was unknown.

## 4. Discussion

This study examined the efficacy and safety of biologic treatments for psoriasis in elderly patients. In accordance with the WHO guidelines [10] we used a cutoff of 65 years to define elderly.

All participants included in the study had experienced failure of at least two conventional systemic therapies, which is a prerequisite in Israel for eligibility for biologic intervention [11]. The adult-start group had a higher rate of failure of cyclosporine than the elderly-start group, probably because cyclosporine is prescribed less often to elderly patients due to its interactions with other agents and necessary dose adjustments.

The presence of comorbid conditions in older patients can influence drug tolerance and safety outcomes [12]. Importantly, we found no significant differences in the prevalence of comorbidities between the adult-start and elderly-start groups, as showen in Table 1, eliminating this factor as a confounder on analysis of the association between age at initiation of biologic therapy and its effects.

In terms of treatment patterns and efficacy, the biologic agents administered most often in our cohort were TNF inhibitors, reflecting their status among the prioritized biologic agents for the treatment of psoriasis by Clalit Health Services. Of the available TNF inhibitors, etanercept is reportedly the most widely used in the elderly [4]. In general, the average duration of treatment with TNF inhibitors was prolonged relative to newer agents. The difference may be attributable to the administration of advanced therapy later in the follow-up period. The number of treatment lines administered was not significantly different between the groups.

All biologic treatments were included in the Kaplan-Meier analysis of drug survival. Previous studies comparing drug survival between elderly and younger psoriatic patients yielded no significant differences, suggesting that drug efficacy and incidence of adverse effects remain consistent across these age groups [4,13,14]. In our cohort, the duration of treatment with adalimumab (as the first line) and with guselkumab (at any sequence) was longer when they were initiated after age 65 years and, for guselkumab, especially after age 75 years. It should be noted that the adult-start group had a significantly longer follow-up period than the elderly-start group, which may have influenced drug survival results, despite the use of censoring in survival analysis.

Reported risk factors for the discontinuation of biologic treatment in all age groups are female gender, obesity, and prior treatments, and in elderly patients, the presence of arthritis [13,15]. In our elderly-start group, 39.7% of patients were obese and 28.9% had psoriatic arthritis. Nevertheless, drug survival for specific drugs was better in this group than in the adult-start group, raising the possibility that there are other factors influencing efficacy, such as the age of treatment initiation.

We observed that the elderly-start group had a significantly better response than the adult-start group to the last treatment recorded during follow-up. The most common agents used at the last treatment in the elderly-start group were ustekinumab followed by guselkumab. Previous studies have shown that both these drugs are associated with an improvement in PASI score over time in elderly patients [16,17].

Our findings are further supported by the real-world data reported by Fiorillo et al., who demonstrated the effectiveness and safety of IL-23 and IL-17 inhibitors in elderly patients with psoriasis [18].

Generally, older patients are at higher risk of adverse events when using systemic medications [7], particularly traditional systemic therapies such as methotrexate and cyclosporine. However, recent studies suggest that certain biologic agents may offer a more favorable safety profile in elderly patients. Borren et al. [19] reported an infection rate of 13% among older patients with immune-mediated diseases. In the present study, the rate of hospitalization for infection in both groups was approximately 16%. However, the time to first hospitalization was relatively long, suggesting that these events may not be directly attributable to biologic therapy protocols. Pneumonia was the predominant cause of hospitalization in both groups, mostly occurring under TNF or IL-23 inhibitor treatment, as previously reported [16,20,21,22,23]. Recent data further support the notion that modern biologic therapies are associated with a relatively low risk of serious infections in elderly patients. Drucker et al. demonstrated that, in older adults with psoriatic disease, the incidence of serious infections was 1.4 per 100 patient-years among those treated with newer biologic agents targeting IL-12, IL-23, or IL-17, compared to 2.5–2.7 per 100 patient-years among those treated with conventional systemic therapies such as methotrexate or cyclosporine [24]. These results suggest that modern biologic therapies may offer a relatively safer infection risk profile in elderly patients, although vigilant monitoring remains essential.

Our findings are further supported by recent real-world studies demonstrating favorable safety and efficacy profiles of IL-23 inhibitors in broader psoriasis populations. For example, Ibba et al. showed sustained effectiveness and safety of risankizumab in patients with and without cardiometabolic comorbidities [25]. In addition, Bellinato et al. concluded that while traditional systemic therapies such as methotrexate and cyclosporine require caution due to increased risk of adverse events in patients with metabolic comorbidities, IL-17 and IL-23 inhibitors appear to offer a safer alternative [26]. Although these studies did not specifically focus on elderly populations, they support the overall safety of these agents in complex clinical scenarios, including patients with multiple comorbidities—a common characteristic in the elderly.

Furthermore, comparative studies have demonstrated that biologic and conventional systemic therapies exhibit similar safety and efficacy profiles between elderly and adult patients with moderate to severe psoriasis, reinforcing the overall safety of biologic therapies in older populations [27]. Although newer biologic therapies appear to be associated with a relatively lower risk of serious infections in elderly patients [24], tolerability issues may still arise with increasing age. Data from the BADBIR registry demonstrated that patients aged 65–74 years were more likely to discontinue TNF inhibitors, IL-12/23 inhibitors, and IL-17 inhibitors compared to younger individuals [28]. Furthermore, patients aged ≥ 75 years were at higher risk of discontinuing any of the four major biologic classes due to adverse events [28] 7/7/2025 9:24:00 a.m. Similarly, a recent multicenter cohort study investigating the impact of age on biologic drug survival in patients with plaque psoriasis reported that elderly patients had an overall higher risk of biologic treatment discontinuation compared to younger adults, particularly when treated with IL-23 inhibitors. However, stratified analyses conducted specifically within the elderly population demonstrated that IL-23 inhibitors maintained higher drug survival rates than IL-17 inhibitors, suggesting a relatively more favorable persistence profile for IL-23 therapies in this age group [29]. These findings underscore the importance of careful patient monitoring and individualized treatment selection in elderly patients receiving biologic therapy.

In parallel, recent systematic reviews and real-world studies have suggested that newer agents, particularly IL-23 inhibitors, may provide a relatively safer treatment option for elderly patients compared to both traditional systemic therapies and older biologics [16,30,31,32]. Our results similarly showed that guselkumab demonstrated superior drug survival in elderly patients, which may reflect favorable safety and tolerability profiles in this population. These findings support the use of biologic therapies as a relatively safe and effective approach in elderly patients with psoriasis, provided that careful patient selection and monitoring are maintained.

There was no difference in the rate of malignancy between our study groups. Prostate carcinoma was the most common malignancy, especially in the elderly-start group, corresponding with the age and sex distribution of the study patients (all aged > 65 years, 63% men) [33]. Accordingly, previous studies found that the rate of malignancies was not different between older and younger patients on biologic therapy, and that there was no disparity in overall cancer diagnosis rates between older individuals using biologics and their non-biologic-using counterparts.18 This stands in contrast to traditional therapies for psoriasis, such as PUVA and conventional systemic treatments, which have previously been associated with an increased risk of malignancy across all age groups [34].

Nonetheless, it should be noted that specific classes of biologic therapies may have different malignancy risk profiles. According to recent data, TNF-α inhibitor therapy in psoriasis was associated with a significantly increased risk of overall cancer and lymphoma, while the risk of solid organ cancers was not elevated. In contrast, IL-12/23 inhibitors have not been associated with an increased risk of any malignancy [35].

Furthermore, an 8-year real-world retrospective study evaluating biological therapy in psoriasis patients with a prior history of malignancy found no increased risk of cancer recurrence or development of new malignancies, supporting the notion that biologic therapies may be safely used even in patients with a history of cancer [36], a finding particularly relevant as the prevalence of prior malignancy increases with age.

Our study did not identify any significant difference in mortality rate between the study groups. The literature describes a higher mortality rate in older than younger users of biologic agents, but no difference among older patients between those using or not using biologic agents [19]. It is noteworthy that within the elderly-start group, infections emerged as the leading cause of death, whereas no patients in the adult-start group died of this cause. Previous literature highlighted the potential association between biologic therapy and an increased risk of infections in older patients. According to our findings, this risk may be influenced by the age at which biologic treatment is initiated but does not necessarily translate into a higher risk of mortality.

Moreover, a recent large cohort study suggested that biologic therapy may even reduce overall mortality risk among patients with psoriasis. However, these findings were not limited to elderly patients [37].

The innovation of this study is comparing the outcome of adult biologic-therapy-naïve patients with psoriasis according to age at initiation of biologic therapy. This unique approach made it possible to comprehensively explore the efficacy, safety, and tolerability of biologic treatments in elderly patients, shedding light on the potential impact of age at initiation on treatment response. By filling this gap in the existing research, our study contributes valuable insights to the understanding of biologic therapy utilization in the elderly population.

This study has several limitations. First, the same specific TNF inhibitors were used in most cases, primarily because of insurance coverage preferences, which could have influenced the results. Second, the retrospective design used has an inherent recall bias. This may have affected the accuracy and completeness of the data, despite our efforts to mitigate this limitation by cross-referencing the information obtained from the patients’ medical records. Additionally, selection bias should be considered, as elderly patients who were prescribed biologic therapy may represent a healthier subgroup, thereby potentially underestimating adverse events in the broader elderly population.

## 5. Conclusions

In conclusion, biologic treatments for psoriasis appear to be both efficacious and safe for use in elderly patients. Certain biologic agents exhibit better drug survival if started after age 65 years. These insights underscore the potential benefits of tailoring treatment strategies to the individual patient based on characteristics such as age at drug initiation and treatment history in order to optimize outcomes in the elderly.

## Figures and Tables

**Table 1:** Baseline demographic and clinical characteristics for each group.

Variable		Elderly-Start Group (≥65 y) *n* = 83	Adult-Start Group (<65 y) *n* = 66	*p* Value
Demographics				
Patient years		695.6	784.3	0.86
Age at the end of follow-up period		79.4 ± 6.3	72.2 ± 3	
Age at diagnosis		56.8 ± 13.3	47.4 ± 10.5	
Sex	Male	49 (59%)	45 (68.1%)	0.25
	Female	34 (40.9%)	21 (31.8%)	
Age at onset of biologic treatment		70.3 ± 4.9	Age 65–70: 50 (60.2%)	59.7 ± 3.6
			Age 70–75: 17 (20.2%)	
			Age > 75: 16 (19.2%)	
Disease duration (years)		21.9 ± 11.7	24 ± 10.5	0.06
Follow-up time (years)		8.3 ± 3.6	11.8 ± 4.1	0.00
Comorbidities				
Weight (kg)		81.8 ± 15.1	82.3 ± 17.8	0.97
BMI (kg/m^2^)		28.9 ± 4.9	28.4 ± 5.1	0.59
Obese				
(BMI * > 30)		33 (39.7%)	24 (36.3%)	0.58
Diabetes mellitus		36 (43.3%)	29 (43.9%)	0.99
Hyperlipidemia		69 (83.1%)	50 (75.7%)	0.2
Hypertension		55 (66.2%)	41 (62.1%)	0.53
History of malignancy		15 (18%)	9 (13.6%)	0.44
Psoriatic arthritis		24 (28.9%)	29 (43.9%)	0.05
History of psoriasis treatment failure				
Methotrexate		51 (61.44)	47 (71.21)	0.21
Acitretin		58 (69.87)	50 (75.75)	0.42
Cyclosporine		1 (1.20)	9 (13.63)	0.005
Phototherapy		82 (98.79)	63 (95.45)	0.79

Baseline demographic and clinical characteristics of the study population. Data are presented for two groups: the elderly-start group (≥65 years, *n* = 83) and the adult-start group (<65 years, *n* = 66). Values are expressed as mean ± standard deviation (SD) or number of patients and percentage, as appropriate. * BMI, body mass index.

**Table 2:** Efficacy of biologic treatment (perceny PASI change).

Variable		Elderly-Start Group (≥65 y) *n* = 83	Adult-Start Group (<65 y) *n* = 66	*p* Value
Initial disease severity by mean PASI *		21.3	22.7	0.17
Disease severity after first biologic treatment (delta PASI)	<50	47 (62.6%)	39 (68.4%)	0.84
	≥50	17 (22.7%)	16 (28.1%)	
	100	11 (14.7%)	2 (3.5%)	
Disease severity after final biologic treatment (delta PASI)	<50	4 (5.1%)	18 (29%)	0.003
	≥50	27 (34.6%)	18 (29%)	
	100	47 (60.3%)	26 (41.9%)	

Efficacy of biologic treatment in the elderly-start group (≥65 years, *n* = 83) and the adult-start group (<65 years, *n* = 66). The table presents changes in Psoriasis Area and Severity Index (PASI) following biologic treatment. Disease severity is assessed at treatment initiation, after the first biologic treatment, and after the final biologic treatment. PASI improvement is categorized as <50% reduction, ≥50% reduction, or 100% clearance. Values are presented as *n* (%) or mean PASI scores. A significant difference was observed between the groups after the final biologic treatment, with a higher proportion of elderly-start patients achieving complete clearance (PASI 100), (*p* = 0.003). * PASI, Psoriasis Area and Severity Index.

## Data Availability

The data that support the findings of this study are not openly available due to reasons of sensitivity and are available from the corresponding author upon reasonable request. Data are in controlled access data storage at Rabin Medical Center—Beilinson Hospital.

## Supplementary Materials

The following supporting information can be downloaded at: https://www.mdpi.com/article/10.3390/jcm14134779/s1, Figure S1: Drug survival of guselkumab in any sequence of treatments in sub-groups of elderly-start and adult-start groups; Table S1: Types of infections acquired while under biologic treatment; Table S2: All agents administrated in each treatment line.

## Abbreviations

The following abbreviations are used in this manuscript:

IL	Interleukin
PASI	Psoriasis Area and Severity Index
TNF	Tumor necrosis factor
