# Supplementary material for: Systemic Biologic Treatment for Psoriasis in Elderly Patients
Source: J Clin Med. 2025 Jul 7;14(13):4779. doi: 10.3390/jcm14134779 (PMC12251099; doi:10.3390/jcm14134779)
Supplement: Supplementary file 1 [file jcm-14-04779-s001.zip › jcm-3706069-supplementary.pdf]

Journal of Clinical Medicine

Original Article

Systemic Biologic Treatment for Psoriasis in Elderly Patients

Sapir Glazer Levavi, MD [1], Ronny Maman, MD [2], Shany Sherman, MD [1,2], Daniel Mimouni, MD [1,2], Lev Pavlovsky, MD, PhD [1,2]

[1] Division of Dermatology, Rabin Medical Center, Petach Tikva, Israel.
[2] Faculty of Medical and Health Sciences, Tel Aviv University, Tel Aviv, Israel.
* Correspondence: Lev Pavlovsky, MD, PhD. Rabin Medical Center, Beilinson Hospital, Petach Tikva 49100, Israel. Email: levpav@gmail.com.

Supplementary materials

Figure S1. Drug survival of guselkumab in any sequence of treatments in sub-groups of elderly-start and adult-start groups.

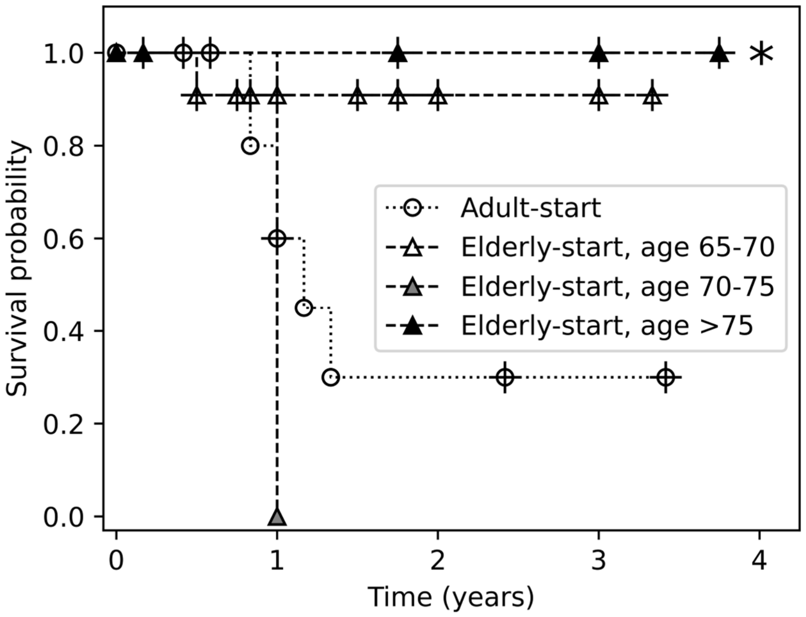

This figure presents the Kaplan-Meier survival curve illustrating drug survival over time. The x-axis represents time (e.g., years), while the y-axis shows the cumulative probability of drug survival. Each line corresponds to a different group, with markers indicating specific data points (e.g., the adult-start group is represented by circles, while the elderly-start subgroups are marked with triangles: unfilled triangles for ages 65–70, gray-filled triangles for ages 70–75, and black-filled triangles for ages 75 and above). Censored events are indicated by vertical tick marks, which represent patients who discontinued treatment due to death, transfer to a different healthcare provider, emigration from Israel, or the end of the follow-up period. Key comparisons between groups are shown, highlighting significant differences in drug survival rates, with guselkumab in any treatment sequence showing better drug survival in patients over 75 years old (p = 0.046).

 https://doi.org/10.3390/jcm14134779

Table S1. All agents administrated in each treatment line.

**Agents / Line of treatment**	Etanercept	Adalimumab	Infliximab	Ustekinumab	Secukinumab	Ixekizumab	Guselkumab	Risankizumab
**Elderly-start group (≥65y) n=83**								
1	52	22	0	6	2	0	1	0
2	10	28	0	11	4	3	5	1
3	0	2	0	18	8	2	3	4
4	0	0	0	3	8	6	4	0
5	0	0	1	1	0	2	4	0
6	0	0	0	0	0	0	1	1
7	0	0	0	0	0	0	0	0
8	0	0	0	0	0	0	0	0
**Adult-start group (<65y, n=66)**								
1	47	11	3	4	1	0	0	0
2	6	31	6	8	1	1	1	1
3	3	5	4	18	8	0	2	1
4	0	1	2	7	10	7	2	1
5	0	1	0	0	3	7	2	7
6	0	0	1	0	0	1	3	3
7	0	0	0	0	0	0	2	2
8	0	0	0	0	0	0	0	1

Biologic agents administered in each treatment line for elderly-start (≥65 years, n=83) and adult-start (<65 years, n=66) psoriasis patients. The table displays the number of patients receiving each biologic agent at each line of treatment. The first-line treatments were most commonly etanercept and adalimumab in both groups. Some patients required multiple lines of treatment, with the maximum number of biologic treatment lines reaching six in the elderly-start group and eight in the adult-start group.

Table S2. Types of infections acquired while under biologic treatment.

Drug	Elderly-start group (≥65y) n=83	Adult-start group (<65y) n=66
Etanercept	soft tissue infections (2)<br>herpes zoster infection (1)<br>gastroenteritis (1)<br>fever (1)	fever (1)
Adalimumab	pneumonia (2)<br>septic shock (1)<br>urosepsis (1)<br>necrotizing fasciitis (1)<br>chronic osteomyelitis (1)	pneumonia (1)<br>gastroenteritis (1)<br>cholecystitis (1)
Infliximab	pneumonia (1)	
Ustekinumab	pneumonia (3)<br>urosepsis (1)<br>urinary tract infection (1)<br>fever (1)	pneumonia (2)<br>sepsis (1)<br>acute osteomyelitis (1)<br>soft tissue infections (1)
Secukinumab		necrotizing fasciitis (1)
Ixekizumab		gastroenteritis (1)<br>urinary tract infection (1)
Guselkumab	cholangitis (1)<br>COVID19 (1)	septic shock (1)
Risankizumab	sepsis (1)<br>COVID19 (1)<br>fever (1)	septic arthritis (1)

Types of infections acquired while under biologic treatment in elderly-start (≥65 years, n=83) and adult-start (<65 years, n=66) psoriasis patients. The table presents infection types categorized by the specific biologic drug used. The most common infection overall was pneumonia, occurring in 9 patients (6% of all patients in both study groups).
